# ROR2 suppresses metastasis of prostate cancer via regulation of miR-199a-5p–PIAS3–AKT2 signaling axis

**DOI:** 10.1038/s41419-020-2587-9

**Published:** 2020-05-15

**Authors:** Jen-Chih Tseng, Shih-Han Huang, Ching-Yu Lin, Bi-Juan Wang, Shiu-Feng Huang, Ying-Ying Shen, Chih-Pin Chuu

**Affiliations:** 1 Institute of Cellular and System Medicine, National Health Research Institutes, Miaoli County, 35053 Taiwan; 2 Department of Life Sciences, National Central University, Taoyuan City, 32001 Taiwan; 3 Institute of Molecular and Genomic Medicine, National Health Research Institutes, Miaoli County, 35053 Taiwan; 4 Pathology Core Lab, National Health Research Institutes, Miaoli County, 35053 Taiwan; 5 PhD Program for Aging and Graduate Institute of Basic Medical Science, China Medical University, Taichung City, 40402 Taiwan; 6 Biotechnology Center, National Chung Hsing University, Taichung City, 40227 Taiwan

**Keywords:** Metastasis, Tumour-suppressor proteins, Prostate cancer, Cell migration, Cell signalling

## Abstract

Bones are the most common metastatic sites for prostate cancer (PCa). Receptor tyrosine kinase-like orphan receptor 2 (ROR2), a noncanonical Wnt receptor, plays crucial roles in skeletal morphogenesis, osteoblast differentiation, and bone formation. The role of ROR2 in PCa metastasis is unclear. We analyzed online datasets from Oncomine as well as using IHC staining on tissue array to determine the relationship between ROR2 expression level and disease outcome of PCa. To investigate how ROR2 regulates migration and invasion of PCa cells, we performed transwell assay and orthotopic xenograft model in nude mice. We then applied the Micro-Western Array (MWA), a high-throughput western blotting platform to analyze the downstream signaling pathways being regulated by ROR2. Compared with nonmalignant PZ-HPV-7 and RWPE-1 cells, PCa cell lines express lower level of ROR2 protein. Constitutive expression of ROR2 in PC-3, DU-145, or C4-2B PCa cells significantly suppressed the cell migration, invasion, and epithelial–mesenchymal transition (EMT) proteins. MWA, western blotting, and microRNA analysis showed that elevation of ROR2 suppressed the expression of miR-199a-5p, which in turn increased the expression of PIAS3. The upregulation of PIAS3 then decreased AKT2 and the phosphorylation of AKT, resulting in the inhibition of migration and invasion of PCa cells both in vitro and in orthotopic xenograft mice model. IHC staining of tissue array and Oncomine datasets analysis indicated that the gene and protein level of ROR2 is much lower in metastatic prostate tumors as compared with primary tumors or adjacent normal prostate tissues. Low level of ROR2 correlated to poor survival and high recurrent frequency in PCa patients. In conclusion, we discovered that ROR2 suppresses PCa metastasis via regulation of PIAS3–PI3K–AKT2 signaling axis.

## Introduction

Bones and lymph nodes are the most common metastatic sites for prostate cancer (PCa). Approximately 90% of patients with advanced PCa have skeletal lesions^1^. PCa metastases to bone are osteoblastic and induce extensive new bone deposition^1^. Wnt signaling plays essential role in bone metastasis of PCa. Canonical Wnt signaling transduces via Frizzled and LRP5/6 receptors to the β-catenin signaling and therefore regulates the cell fate and proliferation^2^. On the other hand, noncanonical Wnt signaling transduces through Frizzled or receptor tyrosine kinase-like orphan receptor (ROR) to the planar cell polarity, G protein-coupled receptor, and receptor tyrosine kinase signaling cascades to regulate cytoskeletal dynamics and directional cell movement^2^. PCa cells produce a Wnt signaling inhibitor DKK-1 in the early stage of skeletal metastases, which masks osteogenic Wnts and causes osteolytic bone degradation at the metastatic site. DKK-1 expression is inhibited during the metastases progresses, allowing the initiation of Wnt-mediated osteoblast^3^.

Receptor tyrosine kinase-like orphan receptor 2 (ROR2) plays crucial roles in skeletal morphogenesis and promotes osteoblast differentiation and bone formation^4^. ROR2 binds with ligand Wnt5a and activates the noncanonical Wnt pathway by activating the Wnt–JNK pathway and inhibiting the β-catenin–TCF pathway^5,6^. Functional role of ROR2 in cancer progression depends on cancer types^7^. ROR2 is epigenetically inactivated in the early stages of colorectal cancer and the reduction of ROR2 contributes to the promotion of Wnt signaling, tumor growth, and cancer metastasis^8,9^. In contrast, the activation of ROR2 with Wnt5a in melanoma cells enhances cell migration and elevates drug resistance to BRAF inhibitors under hypoxia condition^10^. Suppression of ROR2 decreases expression level of MMP-2, and inhibits cell migration and tumor growth in renal cancer^11,12^. However, the role of ROR2 in PCa metastasis is not clear. We therefore introduced Micro-Western Array (MWA), a high-throughput antibody-based proteomics platform^13^, and orthotopic animal model to unravel the role of ROR2 in PCa metastasis.

## Results

### Gene expression of ROR2 is lower in metastatic prostate cancer

**Fig. 1:**
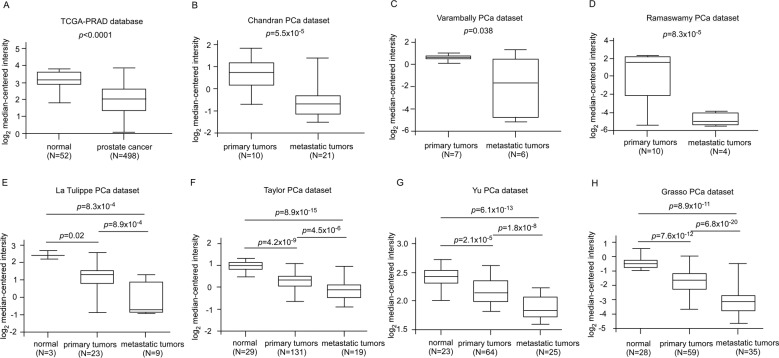
Gene expression level of *ROR2* is higher in adjacent normal prostate tissues as compared with primary prostate tumors and is lowest in metastatic prostate tumors. Gene expression level of *ROR2* in adjacent normal prostate tissues, primary prostate tumors, and metastatic prostate tumors was analyzed in (**a**) TCGA–PRAD database (52 normal prostate tissues, 498 primary prostate tumors), (**b**) Chandran Prostate dataset (10 primary prostate tumors, 21 metastatic prostate tumors), (**c**) Varambally Prostate dataset (7 primary prostate tumors, 6 metastatic prostate tumors), (**d**) Ramaswamy Multi-Cancer dataset-1 (10 primary prostate tumors, 4 metastatic prostate tumors), (**e**) La Tulippe Prostate dataset (3 adjacent normal prostate tissues, 23 primary prostate tumors, and 9 metastatic prostate tumors), (**f**) Taylor Prostate dataset (29 adjacent normal prostate tissues, 131 primary prostate tumors, and 19 metastatic prostate tumors), (**g**) Yu Prostate dataset (23 adjacent normal prostate tissues, 64 primary prostate tumors, and 25 metastatic prostate tumors), (**h**) Grasso (28 adjacent normal prostate tissues, 59 primary prostate tumors, and 35 metastatic prostate tumors) dataset. Statistical significance was shown by the *p* value between the two groups being compared.

To investigate the role of ROR2 in cancer, we first examine the gene expression level of *ROR2* in different types of cancer using the Oncomine database (Supplementary Fig. 1). We noticed that the expression of ROR2 is downregulated in PCa, bladder cancer, brain cancer, head and neck cancer, and ovarian cancer, while ROR2 is upregulated in pancreatic cancer, myeloma, sarcoma, and breast cancer. These observations suggested that ROR2 is a potential tumor suppressor in PCa. We further analyzed *ROR2* gene expression level in 135 adjacent normal prostate tissues, 812 primary prostate tumors, and 122 metastatic prostate tumors from The Cancer Genome Atlas (TCGA) and Oncomine databases. All datasets revealed that prostate tumors express lower *ROR2* gene level as compared with adjacent normal prostate tissues, while metastatic prostate tumors express the lowest *ROR2* level (Fig. 1a–h). Analysis of *ROR2* mRNA expression in human PCa tissue cDNA array with qRT-PCR uncovered that *ROR2* gene level was significantly lower in prostate tumors with Gleason score > 7 as compared with that in adjacent normal prostate tissues or prostate tumors with Gleason score ≦ 7 (Supplementary Fig. 2).

### ROR2 suppresses the migration and invasion of PCa cells

**Fig. 2:**
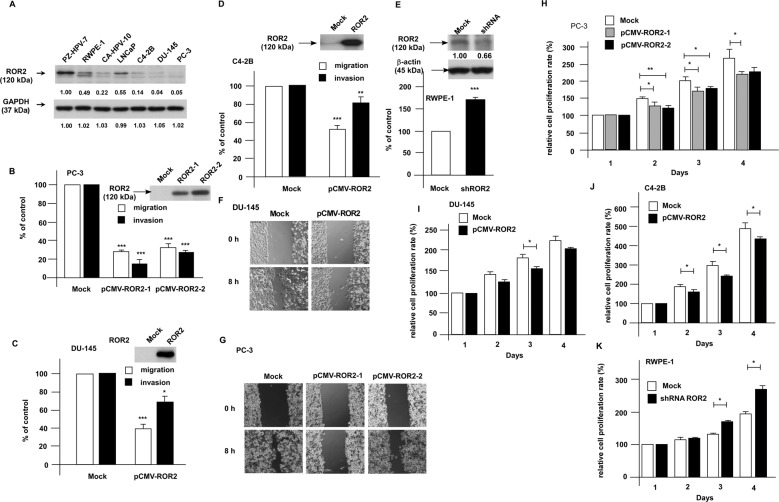
Elevation of ROR2 suppressed migration and invasion of PCa cells. (**a**) ROR2 protein level in nonmalignant PZ-HPV-7, RWPE-1 cells prostate epithelial cells, and commonly used PCa cell lines (CA-HPV-10, LNCaP, LNCAP C4-2B, DU-145, PC-3) was examined by western blot. GAPDH was used as loading control. Cell migration and invasion of control PC-3 cells and PC-3 cells overexpressing ROR2 (pCMV-ROR2-1 and pCMV-ROR2-2) (**b**), control DU-145 cells and DU-145 cells overexpressing ROR2 (**c**), control C4-2B cells and C4-2B cells overexpressing ROR2 (**d**), as well as control RWPE-1 cells and RWPE-1 cells with ROR2 shRNA knockdown (**e**) were determined by the transwell assay. Asterisks *, **, and *** represent statistically significant *p* < 0.05, *p* < 0.01, and *p* < 0.001, respectively, between the two groups being compared. Cell migration of PC-3 (**f**) or DU-145 (**g**) cells with or without ROR2 overexpression was also examined by wound healing assay. Images were obtained by live imaging microscope (Leica AF 6000 LX, Leica, Wetzlar, Germany). The Effect of the overexpression of ROR2 on proliferation of PC-3 (**h**), DU-145 (**i**), and C4-2B (**j**) PCa cells as well as effect of shRNA knockdown of ROR2 on RWPE-1 (**k**) cells for 24, 48, 72, and 96 h was determined by the proliferation assay. Asterisks * and ** represent statistically significant *p* < 0.05 and *p* < 0.01, respectively, between the two groups being compared.

To further investigate if ROR2 is a tumor suppressor in PCa, we examined the expression level of ROR2 in PZ-HPV-7 and RWPE-1 nonmalignant human prostatic epithelial cell lines and commonly used PCa cell lines. Compared with PZ-HPV-7 and RWPE-1 cells, ROR2 protein level in CA-HPV-10, LNCAP, C4-2B, PC-3, and DU-145 cells was 50–95% less (Fig. 2a, Supplementary Fig. 3). Since C4-2B, PC-3, and DU-145 cells have high migration and invasion ability but very low ROR2 protein level, we hypothesized that elevation of ROR2 protein level will hinder the invasion of PCa cells. To test this hypothesis, we overexpressed ROR2 in PC-3, DU-145, and C4-2B cells but knocked down ROR2 in RWPE-1 cells. Elevation of ROR2 suppressed the migration and invasion of PC-3 (Fig. 2b), DU-145 (Fig. 2c), and C4-2B (Fig. 2d) cells. On the other hand, knockdown of ROR2 with shRNA enhanced the migration of RWPE-1 cells (Fig. 2e). Wound healing assay also demonstrated that increase of ROR2 reduced migration ability of DU-145 (Fig. 2f) and PC-3 (Fig. 2g) cells. The reduction of migration and invasion cannot be exclusively explained by the reduction of cell proliferation rate. The migration and invasion assay, which last 6 and 24 h, respectively, revealed a reduction of 20–90% in cell migration and invasion caused by ROR2 overexpression in PC-3, DU-145, and C4-2B cells (Fig. 2b–d) as well as a 70% increase in migration of RWPE-1 cells (Fig. 2e). However, the overexpression of ROR2 only caused a 0–10% reduction of proliferation in PC-3, DU-145, and C4-2B (Fig. 2h–j) cells, while knockdown of ROR2 did not affect proliferation of RWPE-1 (Fig. 2k) at all within 24 h. Our results confirmed that ROR2 suppresses the migration and invasion of PCa cells.

### Elevation of ROR2 augments PIAS3 expression, but suppresses the expression of EMT proteins, phospho-AKT, NF-κB p65, and STAT3

**Fig. 3:**
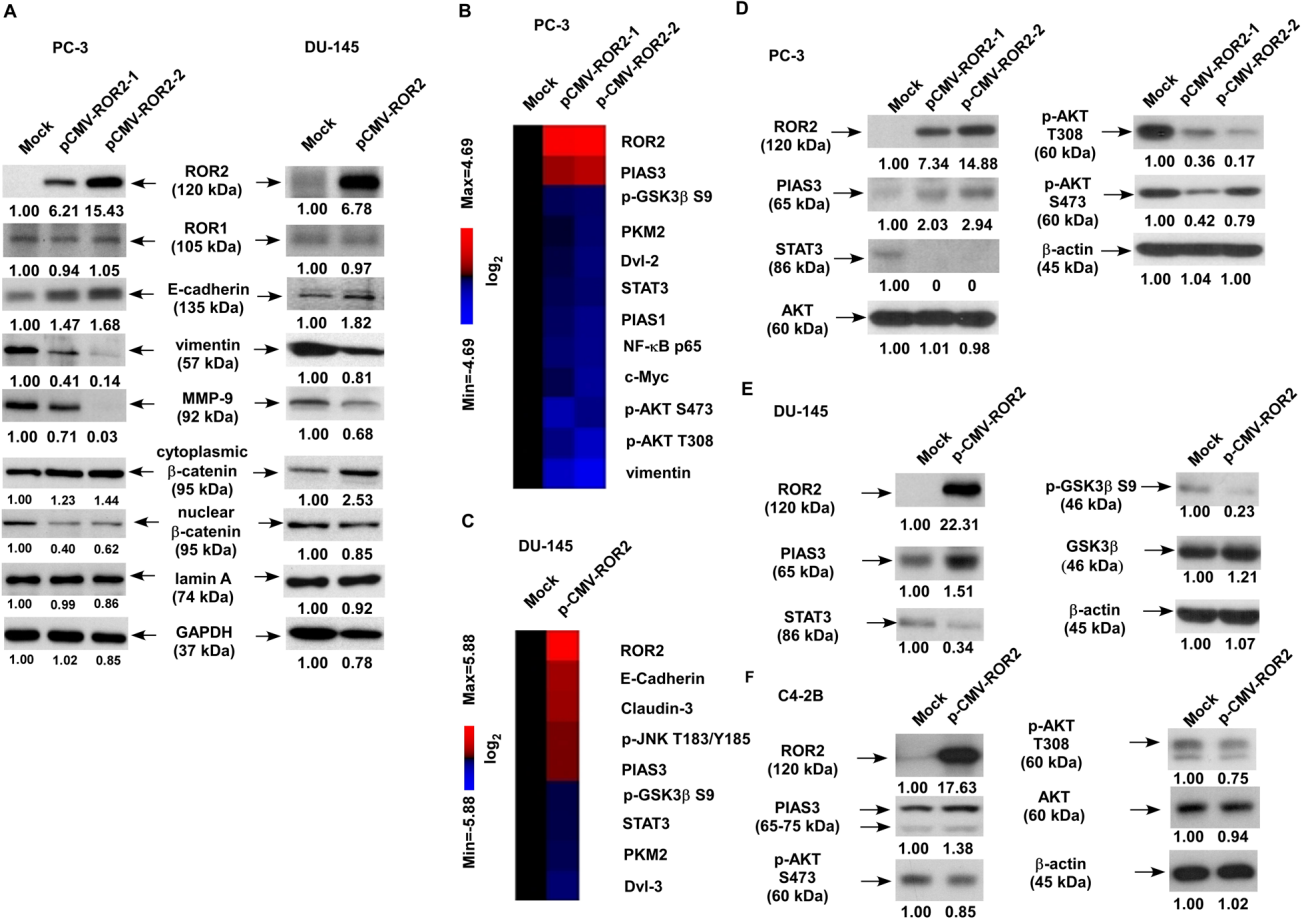
Elevation of ROR2 enhanced PIAS3 expression but suppressed expression of PIAS3, STAT3, and PI3K–AKT signaling and EMT regulatory proteins. (**a**) Expression level of ROR2, ROR1, E-cadherin, vimentin, MMP-9 as well as cytoplasmic and nuclear β-catenin in PC-3 or DU-145 cells with or without ROR2 overexpression were determined by western blotting. Expression of Lamin A/C and GAPDH was used as loading control for nuclear and cytoplasmic protein extract, respectively. Expression levels and phosphorylation status of signaling proteins involved in EMT, Wnt, TGF-β, STAT3, NF-κB, and PI3K–AKT signaling in DU-145 and PC-3 cells with and without ROR2 overexpression were determined by Micro-Western Array (MWA) using 96 different antibodies. Proteins with fold change of increase at least 2-fold or decrease at least 0.5-fold in PC-3 cells (**b**) or DU-145 (**c**) with and without ROR2 overexpression were displayed with heatmap. Expression of β-actin, GAPDH, and α-tubulin was used as loading control. Protein expression levels of ROR2, PIAS3, STAT3, phospho-GSK-3β S9, AKT, phospho-AKT S473, and phospho-AKT T308 in PC-3 (**d**), DU-145 (**e**), and C4-2B (**f**) cells with or without ROR2 overexpression was determined by the western blotting assay. Expression of β-actin was used as loading control.

Elevation of ROR2 increased protein expression of E-cadherin and cytoplasmic β-catenin but decreased protein abundance of vimentin, MMP-9, and nuclear β-catenin in PCa cells (Fig. 3a). These observations suggested that ROR2 suppressed epithelial–mesenchymal transition (EMT), migration and invasion in PCa cells. To investigate the molecular mechanism how ROR2 regulates PCa metastasis, we performed MWA with 96 antibodies targeting metastasis-related signaling pathways to study the signaling network being affected by ROR2 in PC-3 and DU-145 cells with or without ROR2 overexpression (Supplementary Fig. 4). In PC-3 cells, the overexpression of ROR2 significantly increased the expression of protein inhibitor of activated STAT3 (PIAS3), but decreased protein abundance of signal transducer and activator of transcription 3 (STAT3), vimentin, phospho-AKT T308, phospho-AKT S473, c-Myc, NF-κB p65, Dvl-2, PKM2, and phospho-GSK-3β S9 (Fig. 3b). In DU-145 cells, the overexpression of ROR2 significantly increased protein abundance of E-cadherin, Claudin3, phospho-JNK T183/Y185, and PIAS3, but reduced the protein level of Dvl-3, PKM2, STAT3, and phospho-GSK-3β S9 (Fig. 3c). Western blotting analysis confirmed the induction of PIAS3 and the reduction of phospho-AKT S473, phospho-AKT T308, phospho-GSK-3β S9, NF-κB p65, and STAT3 in PC-3, DU-145, and C4-2B cells overexpressing ROR2 (Fig. 3d–f).

### Elevation of ROR2 suppresses metastasis of PC-3 xenografts in orthotopic mice model

**Fig. 4:**
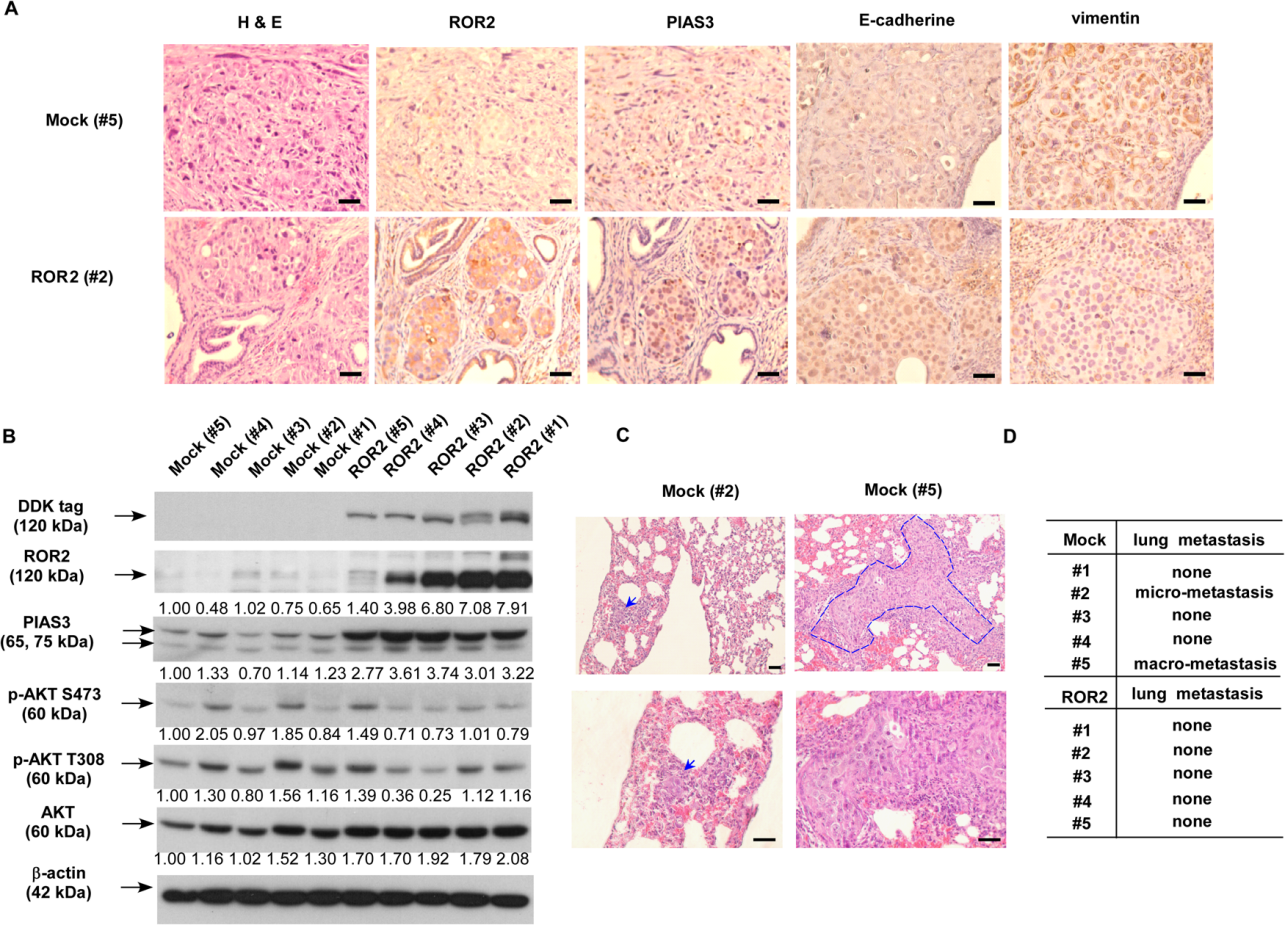
Overexpression of ROR2 suppressed cancer metastasis of PC-3 xenografts in orthotopic nude mice model. (**a**) PC-3 human PCa cells (1 × 10^6^) were directly injected into prostate organ of nude mice to form prostate tumors. There were five mice in control group and experimental group, respectively. Protein expression level of ROR2, PIAS3, E-cadherin, and vimentin was examined by IHC. Images were captured at ×40 magnification. Scale bar represented 50 μm. (**b**) Proteins level of ROR2, PIAS3, phospho-AKT S473, phospho-AKT T308, and total AKT in prostate xenografts were also determined by the western blotting assay. Expression of β-actin was used as loading control. (**c**) Hematoxylin and eosin staining of lung tissues in mice was examined for dissemination of PCa cells. Images were capture at ×20 (upper panel) and ×40 (bottom panel) magnification. Scale bar represented 50 μm. (**d**) A list of lung metastasis of PC-3 cells with or without ROR2 overexpression in the above orthotopic nude mice.

To determine if elevation of ROR2 expression can suppress metastasis of PCa cells in vivo, we injected control PC-3 cells and PC-3 cells stably overexpressing ROR2 orthotopically into the prostates of nude mice. After 3 months, mice were sacrificed and the morphology of prostate xenografts were examined by hematoxylin and eosin (H&E) staining (Supplementary Fig. 5). Overexpression of ROR2 increased the expression of PIAS3 and E-cadherin, but repressed that of vimentin as determined by IHC staining (Fig. 4a). Western blotting demonstrated that overexpression of ROR2 in prostate tumors significantly increased the expression of PIAS3 protein and tended to decrease the abundance of phosphorylation of AKT on S473 and T308 (Fig. 4b). In addition, elevation of ROR2 reduced the metastasis of PC-3 cells from prostate to lung in nude mice (Fig. 4c, d and Supplementary Fig. 6).

### ROR2 suppresses the migration of PCa cells via induction of PIAS3

**Fig. 5:**
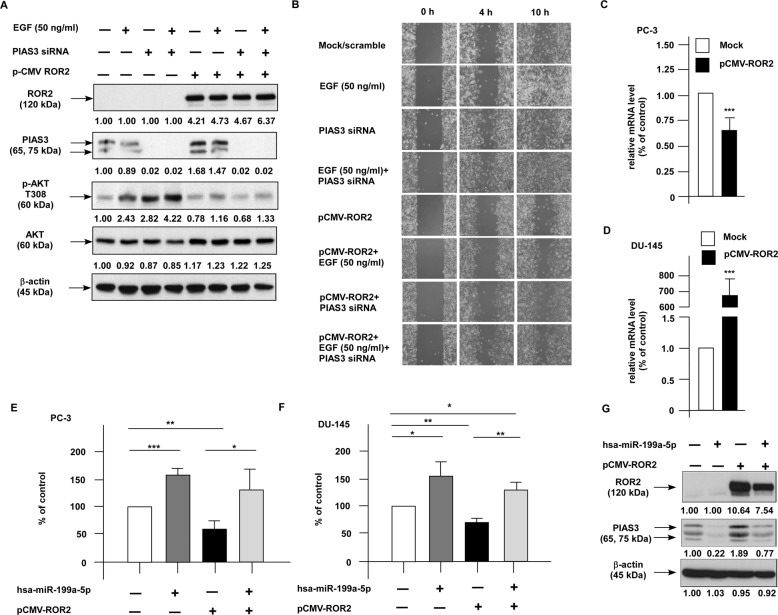
Elevation of ROR2 suppressed PI3K–AKT signaling via activation of PIAS3. (**a**) Proteins expression level of ROR2, PIAS3, phospho-AKT S473, phospho-AKT T308, and total AKT in PC-3 cells with or without ROR2 overexpression and PIAS3 siRNA knockdown in the presence or absence of 50 ng/ml EGF for 24 h treatment was determined by the western blotting assay. Expression of β-actin was used as loading control. (**b**) Cell migration of PC-3 cells with or without ROR2 overexpression and PIAS3 siRNA knockdown in the presence or absence of 50 ng/ml EGF for 24 h treatment was determined by the wound healing assay. Expression level of *hsa-miR-199a-5p* in control PC-3 cells or PC-3 overexpressing ROR2 cells (**c**) or in control DU-145 cells or DU-145 overexpressing ROR2 cells (**d**) was determined by qRT-PCR. Expression of *U6* snRNA was used as loading control. PC-3 (**e**) or DU-145 (**f**) cells transfected with control vector or pCMV-ROR2 vector with or without overexpression of *miR-199a-5p* were cultured for 72 h. Migration ability of these PCa cells was determined by transwell assay. Asterisks *, **, and *** represent statistically significant difference *p* < 0.05, *p* < 0.01, and *p* < 0.001, respectively, between the two groups being compared. (**g**) Expression level ROR2 and PIAS3 proteins in PC-3 cells transfected with control vector or pCMV-ROR2 vector with or without overexpression of *miR-199a-5p* was determined by the western blotting assay. The β-actin was used as loading control.

Upregulation of PI3K (phosphoinositide 3-kinase)–AKT signaling was commonly detected in the prostate tumors, resulting in an increase of tumor growth and cancer metastasis^14,15^. PIAS3 is an endogenous inhibitor for activated STAT3^16^, and it is involved in the regulation of signaling protein function such as NF-κB and PI3K–AKT signaling^17–19^. As overexpression of ROR2 significantly increased PIAS3 (Fig. 3, Fig. 4) and the gene expression level of PIAS3 slightly positively correlated to the gene expression level of ROR2 (Supplementary Fig. 7) in TCGA–PRAD database, we investigated if elevation of ROR2 inhibits migration and invasion of PCa cells via induction of PIAS3. EGF treatment increased phosphorylation of AKT and migration of PC-3 cells while overexpression of ROR2 suppressed the AKT phosphorylation as well as reduced migration of PC-3 cells (Fig. 5a, b). Knockdown of PIAS3 increased phosphorylation of AKT and the migration of PC-3 cells. Knockdown of PIAS3 partially rescued the inhibition of AKT phosphorylation and cell migration caused by ROR2 overexpression (Fig. 5a, b). Activation of PI3K–AKT signaling by adding EGF rescued the suppressive effect of ROR2 on cell migration (Fig. 5a, b).

### ROR2 suppresses the migration of PCa cells via regulation of miR-199a-5p–PIAS3–AKT2 signaling axis

**Fig. 6:**
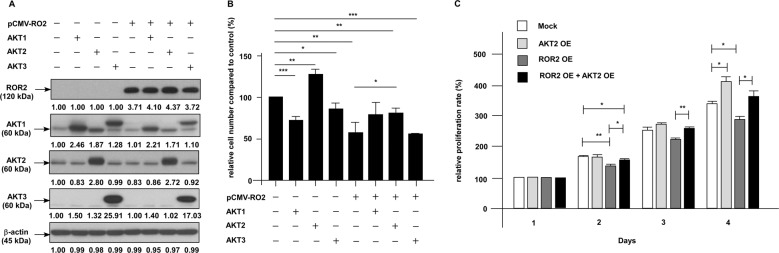
Elevation of ROR2 suppressed migration of PCa cells via inhibition of AKT2. (**a**) PC-3 cells with or without ROR2 overexpression were transfected with plasmid to overexpress either AKT1, AKT2, or AKT3. Cells were cultured for 72 h and the proteins levels of ROR2, AKT1, AKT2, and AKT3 in these PC-3 PCa cells were determined by the western blotting assay to confirm the overexpression of proteins. The β-actin was used as loading control. (**b**) Migration of PC-3 cells with or without ROR2, AKT1, AKT2, and AKT3 overexpression in (**a**) was determined by the transwell assay. (**c**) Proliferation of PC-3 cells with or without ROR2 or AKT2 overexpression in (**b**) was determined by the proliferation assay.

To clarify how ROR2 elevated PIAS3 in PCa cells, we examined whether ROR2 increases the transcription level of *PIAS3*. Overexpression of ROR2 in PC-3 and DU-145 PCa cells did not alter the mRNA level of *PIAS3* (data not shown). As PIAS3 is regulated by miRNAs (http://www.microrna.org), we determined if elevation of ROR2 alters miRNAs expression, resulting in the increase of PIAS3. Expression of *miR-18a*, *miR-143*, *miR-150*, *miR-181a*, *miR-181b*, *miR-181c*, *miR-181d*, *miR-199a-5p*, *miR-200a*, and *miR-613* were significantly suppressed by the overexpression of ROR2 in PC-3 cells (Fig. 5c, Supplementary Fig. 8), while *miR-18a*, *miR-18b*, *miR-141*, *miR-181a*, *miR-181b*, *miR-181c*, *miR-181d*, *miR-185, miR-199a-5p*, *miR-200a*, and *miR-340* were significantly suppressed by the overexpression of ROR2 in DU-145 cells (Fig. 5d, Supplementary Fig. 9). Overexpression of *miR-199a-5p* rescued the inhibition on migration caused by the elevation of ROR2 in PC-3 (Fig. 5e) and DU-145 (Fig. 5f) cells. We determined if alteration of these microRNA can affect the protein abundance of PIAS3. We observed that ROR2 overexpression increased protein level of PIAS3, while overexpression of *miR-199a-5p* compromised the effect of ROR2 on PIAS3 protein abundance in PC-3 cells (Fig. 5g). This result suggested that *miR-199a-5p* is a downstream target of ROR2. Our western blotting demonstrated that elevation of ROR2 in PCa cells significantly increased the expression of PIAS3 protein and decreased the phosphorylation of AKT in PC-3 and C4-2B cells (Fig. 3). As AKT has three isoforms, AKT1, AKT2, and AKT3, we examined which isoform is involved in the regulation of migration of PCa cells overexpressing ROR2. We discovered that overexpression of AKT2, but not AKT1 or AKT3, rescued the inhibitory effect of ROR2 on cell migration of PC-3 cells (Fig. 6a, b). As migration assay was performed for only 6 h and overexpression of AKT2 did not affect proliferation of PC-3 cells within 24 h, the rescue effect of AKT2 was not due to the reduction of cell proliferation (Fig. 6c).

### Protein level of ROR2 is downregulated in metastatic prostate tumors

**Fig. 7:**
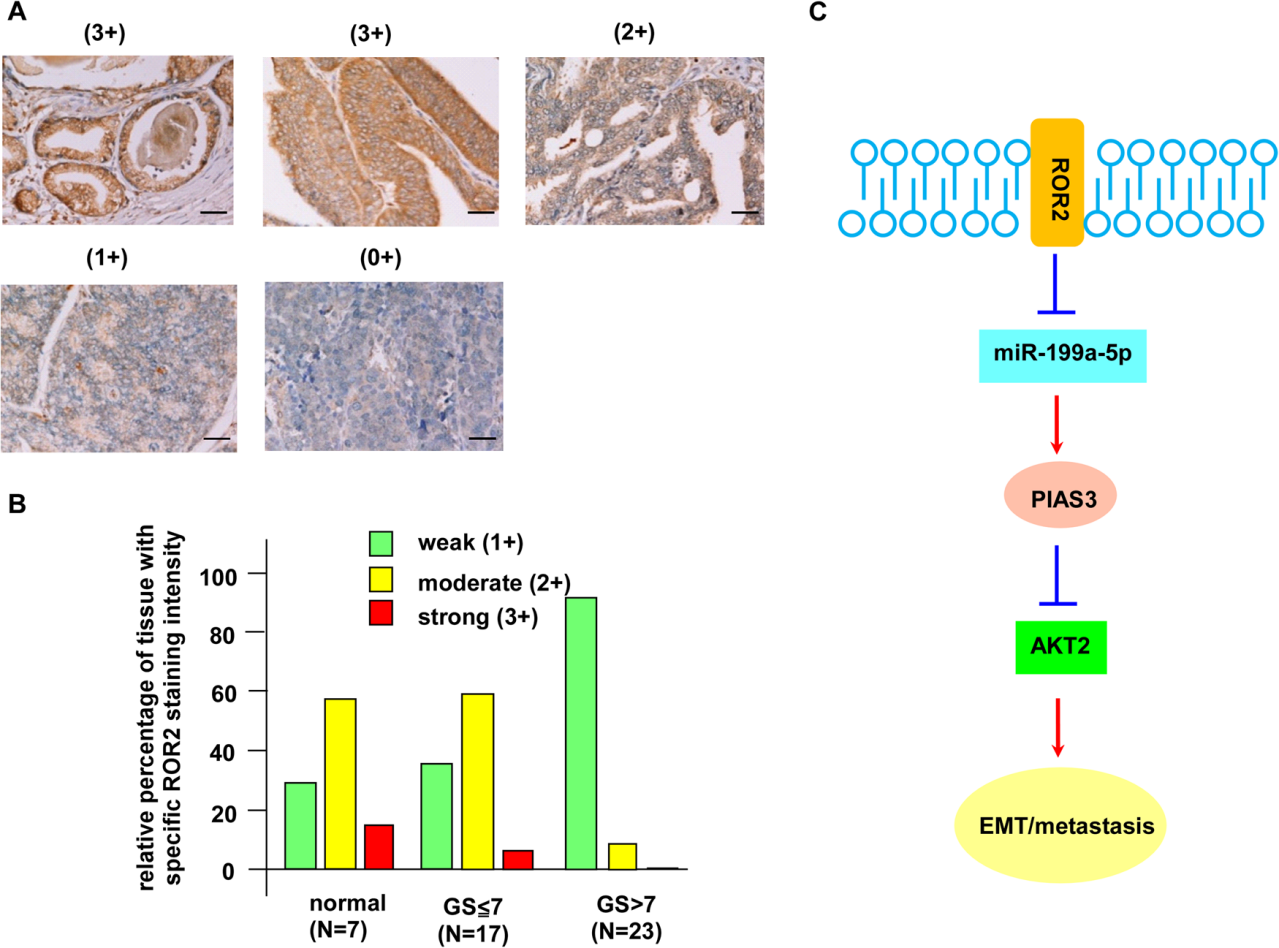
ROR2 protein expression level is downregulated in metastatic prostate tumors. (**a**) ROR2 protein level in prostate tumors was determined by IHC staining with tissue array. The IHC staining intensity of ROR2 expression was scored as 3+ (strong), 2+ (moderate) to 1+ (weak), and 0 (no expression). (**b**) The intensities of ROR2 signaling in normal (*N* = 7) prostate tissues, low Gleason score PCa tissues (≦7, *N* = 17), and high Gleason score PCa tissues (>7, *N* = 23) were separated into weak, moderate, and strong ROR2 IHC staining. (**c**) A summary of signaling pathway regulated by ROR2 in PCa. Red arrows indicated signaling proteins being enhanced by elevation of ROR2, while blue arrows indicated signaling proteins being suppressed by the elevation of ROR2. Scale bar represented 50 μm.

As we demonstrated that ROR2 is a novel tumor suppressor inhibiting the migration and invasion of PCa cells, we predicted that the protein level of ROR2 in advanced prostate tumors should be downregulated. Indeed, immunohistochemistry staining revealed that prostate tumors with Gleason score of 7 express much weaker ROR2 protein as compared with adjacent normal prostate tissues or prostate tumors with Gleason score ≤ 7 (Fig. 7a, b). Analysis of the SurvExpress online database revealed that PCa patients with low gene expression level of ROR2 correlated to poor clinical outcome (Supplementary Fig. 10a) and higher recurrent rate (Supplementary Fig. 10b). The TCGA database showed a trend that PCa patients with lower *ROR2* gene expression level correlate to worse survival, although the *p* value was not smaller than 0.05 (Supplementary Fig. 10c).

## Discussion

In this study, we demonstrated that ROR2 is a novel tumor suppressor inhibiting the PCa metastasis. All PCa datasets from Oncomine indicated that gene expression level of *ROR2* was downregulated in prostate tumors as compared with normal tissues (Supplementary Fig. 1). In patients, the gene and protein level of ROR2 was much lower in primary tumors as compared with that in adjacent normal prostate tissues, while metastatic prostate tumors expressed the least ROR2 gene and protein (Fig. 1). Low level of ROR2 correlated to poor survival and high recurrent frequency in PCa patients (Supplementary Fig. 10). Compared with nonmalignant PZ-HPV-7 and RWPE-1 prostate epithelial cells, PCa cells expressed less ROR2 proteins (Fig. 2). Overexpression of ROR2 in DU-145, PC-3, or C4-2B PCa cells significantly suppressed the cell migration, invasion, and EMT marker proteins, while knockdown of ROR2 with shRNA enhanced the migration of RWPE-1 cells (Fig. 2). Orthotopic xenograft mice model demonstrated that elevation of ROR2 suppressed metastasis of PCa cells and PIAS3 protein (Fig. 4).

MWA and western blotting analysis revealed that elevation of ROR2 increased protein expression of PIAS3, but decreased the abundance of STAT3, NF-κB, and phosphorylation of AKT in PCa cells (Fig. 3). PIAS3 is an E3 SUMO-protein ligase. PIAS3 blocks the DNA-binding activity of STAT3 and inhibits STAT3-mediated gene activation^16^. PIAS3 was found to maintain breast cancer organoids in a noninvasive state via sumoylation of Smurf2 and suppresses breast cancer organoid invasiveness^20^. Expression of PIAS3 is induced by androgen in PCa cells^21^ and PIAS3 enhances the transcriptional activity of AR^22^. We observed that PIAS3 in PCa cells was regulated by *miR-199a-5p* and the increase of PIAS3 suppressed the migration and invasion of PCa cells (Fig. 5). Recently, PIAS3 was reported to suppress cell proliferation and to restore the drug sensitivity of human lung cancer cells by suppressing AKT phosphorylation^19^. Since activation of PI3K–AKT signaling by adding EGF (Fig. 5) or overexpression of AKT2 (Fig. 6) rescued the suppressive effect of ROR2 on PCa cell migration, we concluded that ROR2 suppressed migration, invasion, and EMT of PCa cells via regulation of miR-199-5p/PIAS3/AKT2 axis signaling (Fig. 7c). We noticed that, although overexpression of ROR2 increased PIAS3 in DU-145 cells, it only altered the expression of GSK-3β and phospho-GSK-3β but not AKT or phospho-AKT in DU-145 cells (Fig. 3). Phosphatase and tensin homolog (PTEN) protein is a phosphatase dephosphorylating phosphatidylinositol (3,4,5)-trisphosphate. PTEN is a negative regulator for PI3K–AKT signaling pathway^23^. Deletion of PTEN was observed in 40–70% of PCa patients, resulting in upregulation of PI3K–AKT signaling. PI3K–AKT signaling plays an important role in the survival of PCa cells^24–26^. Upregulation of PI3K–AKT activity is associated with poor clinical outcome of PCa^24,27–30^. The PC-3 and C4-2B cells do not express PTEN, but DU-145 cells express PTEN protein^31^. As a result, activity of AKT is suppressed by PTEN in DU-145 cells, which may explain why overexpression of ROR2 did not significantly suppress AKT and phospho-AKT in DU-145. As GSK-3β is an important downstream signaling protein in PI3K–AKT signaling pathway, ROR2 possibly regulates migration and invasion of DU-145 cells via miR-199a-5p–PIAS3–GSK-3β.

The *miR-199a-5p* is a very conserved miRNAs during the evolution and it plays essential role in the regulation of angiogenesis^32^, cell proliferation^33^, and autophagy^34^. Elevation of *miR-199a-5p* is involved in regulation of cardiomyocyte and endothelial cell function, resulting in terminal failing hearts^35^. The *miR-199a-5p* directly binds to the 3′UTRs of the mRNA of both PIAS3 and p27^Kip1^ and mediates the reduction of PIAS3 and p27^Kip1^ proteins. The *miR-199a-5p* stimulates the STAT3, cell cycle progression, and tumor growth in osteosarcoma cells^36^. The *miR-199a-5p* promotes migration and tube formation of human cytomegalovirus-infected endothelial cells through downregulation of SIRT1 and eNOS^37^. In Addition, *miR-199a-5p* elevates invasion of cutaneous squamous cell carcinoma by suppressing E-cadherin expression^38^. Upregulation of *miR-199a-5p* increased cell invasion and metastasis as well as EMT of gastric cancer cells^39,40^. Our study implied that upregulation of ROR2 suppresses the expression of *miR-199a-5p* and increases the expression of PIAS3 (Fig. 5), possibly through the direct interaction between *miR-199a-5p* and the 3′UTRs of PIAS3 mRNA.

In conclusion, reduction of ROR2 receptor expression in PCa cells increases *miR-199a-5p*, resulting in the decrease of PIAS3. The reduction of PIAS3 then elevates AKT2, therefore enhances EMT and promotes the metastasis of PCa cells. Our results suggested that ROR2 is a novel tumor suppressor in PCa.

## Materials and methods

### Chemicals

All chemicals used in this research were purchased from Sigma-Aldrich (St. Louis, MO, USA).

### Cell culture

RWPE-1, PZ-HPV-7, CA-HPV10, LNCaP FGC, DU-145, and PC-3 were purchased from Bioresource Collection and Research Center (Hsinchu city, Taiwan). LNCAP C4-2B cell line was a gift from Dr Hsing-Jien Kung (NHRI, Taiwan). RWPE-1, PZ-HPV-7, and CA-HPV10 were maintained in Keratinocyte-SFM (Gibco/Thermo Fisher Scientific, Waltham, MA, USA) with bovine pituitary extract and EGF. LNCaP FGC, DU-145, and PC-3 cells were maintained in Dulbecco’s Modified Eagle’s Media (DMEM) containing 10% FBS, penicillin (100 U/ml), and streptomycin (100 μg/ml). LNCAP C4-2B cells were cultured in RPMI-1640 containing 10% FBS. All human cell lines have been authenticated using STR profiling within the last 3 years.

### Quantitative real-time PCR for miRNA

The total RNA was isolated by using the Qiagen RNeasy Mini kit (Qiagen, Venlo, Netherlands), and 2 μg complementary DNA was produced by the RevertAid H Minus First Strand cDNA kit (Thermo Fisher Scientific). The expression level of miRNA was determined by using the qSTAR miRNA qPCR detection system (OriGene) and was normalized with U6 snRNA. All primers used in present study are listed in Supplementary Table 1.

### Plasmids and siRNA

Expression of ROR2-Flag plasmid (pCMV-ROR2, RC215640), hsa-miR-199a-5p (MIR199A1, SC400250) and its control vector (pCMV-Entry, PS100001) were purchased from OriGene. Small interfering RNA for PIAS3 (ON-TARGET plus SMARTpool, L-004164-00-0005) and nonspecific targeting (ON-TARGET plus Nontargeting pool D-001810-10-05) were purchased from Dharmacon (Lafayette, CO, USA). The sequence of the primers is listed in Supplementary Table 1.

### Transient shRNA transfection

Expression of hairpin shROR2 plasmid (pGPU6-RFP-shROR2, target sequence: CCAGCCAAGACATGGAAAT) and its control vector (pGPU6-RFP-shNC, target sequence: TTCTCCGAACGTGTCACGT) purchased from GeneDireX (Taichung City, Taiwan) were used for knockdown of ROR2. For knockdown of ROR2, 5 × 10^5^ RWPE-1 were seeded onto six-well culture plate for overnight. RWPE-1 cells were transfected with 1 μg plasmid using PolyJet DNA In Vitro transfection reagent. The ratio of plasmid and PolyJet was 1 μg DNA to 3 μl transfection reagent. After 6 h of transfection, cells were subculture for additional 24–48 h for further examinations.

### Cell extract and subcellular fractionation

Whole cell lysates used in present study were extracted by TNET buffer (50 mM Tris-HCl, pH 7.5, 150 mM NaCl, 5 mM EDTA and 1% Triton X-100) with 1X proteinase and phosphatase inhibitors cocktail at 4 °C and centrifuged at 15,000 rpm for 30 min. Subcellular fractionation was separated with two steps of differential extraction. Protein samples of cytoplasmic were extracted by using low percentage of NP-40 lysis buffer (50 mM Tris-HCl, pH 7.5, 5 mM MgCl_2_ and 0.4% NP-40) with 1X proteinase and phosphatase inhibitors cocktail at 4 °C. Cytoplasmic lysate and intact of cell nuclei were separated at 4 °C and centrifuged with 6000 rpm for 5 min. The clear suspensions were transferred into new 1.5 ml tubes and pellets were washed once with 0.1% NP-40 buffer. Finally, nuclear part of protein samples were extract by using Buffer C (20 mM HEPES, pH 7.9, 1.5 mM MgCl_2_, 420 mM NaCl, 0.2 mM EDTA, 50% glycerol and 0.5 mM DTT) with 1X proteinase and phosphatase inhibitors cocktail at 4 °C. Nuclear protein samples were collected at 4 °C and centrifuged with 15000 rpm for 30 min.

### Western blotting analysis

The western blotting assay was performed as previously described^41^. All the antibodies used in present study are listed in Supplementary Table 2. Anti-rabbit and anti-mouse IgG secondary antibodies purchased from Santa Cruz Biotechnology. Intensity of bands for different proteins were quantified with Image J software after EPSON stylus TX130 scanning.

### Transwell migration and invasion assay

Migration and invasion assays for PC-3, DU-145, PZ-HPV-7, and C4-2B cells were performed with a transwell kit from Becton Dickinson (Franklin Lakes, NJ, USA). PC-3, DU-145, and C4-2B cells with or without ROR2 overexpression or RWPE-1 cells with or without ROR2 shRNA knockdown were seeded (2 × 10^4^) into DMEM without serum at upper compartment of transwell. The lower compartment of transwell was loaded with DMEM containing 10% FBS. The cell migration and invasion chambers were inserted into the lower compartment and incubated for 24 h. Cells migrated to another side of filter were fixed with cold methanol for 10 min, and then stained with Hematoxylin solution (0.02%) for 30 min. Cells remained on the topside of the filter were removed with a cotton swab. Migration and invasiveness were evaluated by counting the invading cells under a light microscope. All experiments were conducted in triplicates.

### Wound Healing Assay

Wound healing assay for cell migration was performed with culture inserts purchased from ibidi (Martinsried, Germany) according to the manufacturer’s instructions. Briefly, cells were seeded at a concentration of 3.5 × 10^4^/100 μl into ibidi culture inserts in 24-well plate and incubated in 5% CO_2_ incubator at 37 °C overnight. Fill the culture plate with DMEM complete medium and then removed the ibidi culture inserts. Cell migration was monitored once per two hours by photographing with a live imaging microscope (Leica AF 6000 LX, Leica, Wetzlar, Germany). The results of wound closure were displayed by photographs.

### Micro-Western Arrays (MWA)

DU-145 and PC-3 cells were lysed with the MWA lysis buffer (240 mM Tris-acetate pH 6.9, 1% (w/v) SDS, 0.5% glycerol, 5 mM EDTA, and fresh add 1X protease inhibitor cocktail, phosphatase inhibitor cocktail and 1 mM Na_3_VO_4_.). The MWA was conducted as previously described^13^. Detection of α-tubulin and β-actin were used as loading control. Scanned images were obtained by using Odyssey Infrared Imaging System. Intensity of bands for different proteins was quantified with Odyssey 3.0 software. All the antibodies used in present study are listed in Supplementary Table 2.

### Orthotopic xenograft in athymic mice

Animal protocol was reviewed and approved by IACUC of NHRI. PC-3 cells (1 × 10^6^) of with or without overexpression of ROR2 was directly injected into the prostate of BALB/cAnN.Cg-Foxn1nu/CrlNarl mice (purchased from National Laboratory Animal Center, Taiwan) at 6–8 weeks old. There were five mice in control group and experimental group, respectively. Mice were randomly separated into control and experimental group. Mice were sacrificed after 3 months experimental period. Status of tumor growth and dissemination were determined by using H&E staining.

### Immunohistochemistry

Paraffin embedded tissue sections were derived from commercial human PCa tissue array (SUP-CA array from SuperBioChips, Seoul, Korea and PR956a array from US Biomax, Rockville, MD, USA) or tumor bearing mice. Paraffin embedded tissue sections were deparaffinized with xylene, rehydration with graded concentrations of ethanol, and then practice antigen recovery with 10 mM citrate buffer (pH 6.0) via Heat-Induced Epitope Retrieval method, and blocked endogenous peroxidase with 3% H_2_O_2_ in TBS for 20 min. Samples were blocked with Ultra V block (Thermo Fisher Scientific) and incubated with primary antibody at a 1:100 dilution for overnight at 4 °C. The tissue sections were rinsed with TBST three times per 5 min, and then incubated with HRP conjugated antibodies (*N*-Histofine, NICHIREI Biosciences, Tokyo, Japan). Excess antibodies were removed through being rinsed with TBST three times per 5 min, and then tissue specimens were reacted with DAB chromogen and substrate mixture (Thermo Fisher Scientific) for appropriate timing. Immunostaining was visualized after counterstaining with Hematoxylin. Scoring of immunohistochemistry of commercial tissues arrays were evaluated independently by two pathologists. All of antibodies used are listed in Supplementary Table 2.

### Data analysis

Data were presented as mean ± SD of at least three independent experiments or are representative of experiments repeated more than three times. Student’s *t* test (two-tailed, paired) was used to evaluate the statistical significance.

## Supplementary information

Supplemental Figure Legends

Supplemental Material and Methods

Supplemental Table 1

Supplemental Table 2

Supplemental Figure 1

Supplemental Figure 2

Supplemental Figure 3

Supplemental Figure 4

Supplemental Figure 5

Supplemental Figure 6

Supplemental Figure 7

Supplemental Figure 8

Supplemental Figure 9

Supplemental Figure 10
