# Supplementary material for: ROR2 suppresses metastasis of prostate cancer via regulation of miR-199a-5p–PIAS3–AKT2 signaling axis
Source: Cell Death Dis. 2020 May 15;11(5):376. doi: 10.1038/s41419-020-2587-9 (PMC7228945; doi:10.1038/s41419-020-2587-9)
Supplement: Supplementary file 1 — Supplemental Figure Legends [file 41419_2020_2587_MOESM1_ESM.docx]

**Supplemental Figure Legends**

**Supplemental Figure 1. Gene expression level of *ROR2* in different types of cancer.** Analysis of *ROR2* mRNA level in different types of cancer extracted from Oncomine database was shown. Red bars represented that cancer tissues express higher *ROR2* mRNA level compared to adjacent normal tissues. Blue bars represented that cancer tissues express lower *ROR2* mRNA level compared with adjacent normal tissues.

**Supplemental Figure 2. The mRNA level of *ROR2* in prostate tumors with different Gleason score.** Box plots showed relative *ROR2* mRNA level in 18 adjacent normal prostate tissues, 30 benign prostatic hyperplasia (BPH), 45 prostate tumors with Gleason score ≦7, and 13 prostate tumors with Gleason score ＞7 from TissueScan Prostate Tissue qPCR Array HPRT101~103 assayed with qRT-PCR. The mRNA in each well was quantified to gene expression of β-actin. *p* value smaller than 0.05 was considered statistically significant.

**Supplemental Figure 3. Quantification of ROR2 protein expression level in non-malignant human prostate epithelial cell lines and PCa cell lines.** Result of Western blot assay for ROR2 protein expression level in non-malignant PZ-HPV-7, RWPE-1 cells prostate epithelial cells and commonly used PCa cell lines (CA-HPV-10, LNCaP, LNCAP C4-2B, DU-145, PC-3) shown in Fig. 2A was quantified. Asterisks *, ** and *** represented statistically significant *p* < 0.05, *p* < 0.01 and *p* < 0.001 respectively, between the two groups being compared.

**Supplemental Figure 4. Micro-Western Array (MWA) analysis of expression profile of signaling proteins being affected by elevation of ROR2 in PC-3 and DU-145 PCa cells.** Expression levels and phosphorylation status of signaling proteins involved in EMT, Wnt, TGF-β, STAT3, NF-κB and PI3K-AKT signaling in DU-145 and PC-3 cells with and without ROR2 over-expression were determined by Micro-Western Array (MWA) using 96 different antibodies. A representative image of MWA was shown.

**Supplemental Figure 5. Elevation of ROR2 reduced metastasis of prostate xenografts.** PC-3^luc^ cells (1x10^6^) were directly injected into prostate organ of nude mice of 6-8 weeks old. H&E staining was performed to examine the growth of prostate xenografts when mice were sacrificed. Images were captured at 20X magnification. Scale bar represented 50 μm.

**Supplemental Figure 6. Elevation of ROR2 reduced metastasis of prostate xenografts.** PC-3^luc^ cells (1x10^6^) were directly injected into prostate organ of 6-8 weeks old nude mice. H&E staining was performed to examine lung metastasis of PC-3 PCa cells when mice were sacrificed. Images were captured at 20X magnification. Scale bar represented 50 μm.

**Supplemental Figure 7. Correlation between *PIAS3* and *ROR2* in TCGA-PRAD database.** Correlation between gene expression level of *PIAS3* and *ROR2* in prostate tumors was analyzed using TCGA-PRAD database containing N=498 samples. Pearson correlation coefficient (Pearson's r value) was 0.094 with a *p* value of 0.037.

**Supplemental Figure 8.** **Elevation of ROR2 suppressed expression of microRNA in PC-3 cells.** Expression levels of *hsa-miR-18a*, *hsa-miR-18b*, *hsa-miR-141*, *hsa-miR-143*, *hsa-miR-150*, *hsa-miR-181a*, *hsa-miR-181b*, *hsa-miR-181c*, *hsa-miR-181d*, *hsa-miR-185*, *hsa-miR199a-5p*, *hsa-miR-200a*, *hsa-miR-383*, *hsa-miR-496*, and *hsa-miR-613* in PC-3 cells transfected with control vector or pCMV-ROR2 vector were determined by RT-qPCR. Expression of *U6* snRNA was used as loading control.

**Supplemental Figure 9.** **Elevation of ROR2 suppressed expression of microRNA in DU-145 cells.** Expression levels of *hsa-miR-1, hsa-miR-18a*, *hsa-miR-18b*, *hsa-miR-141*, *hsa-miR-150*, *hsa-miR-181a*, *hsa-miR-181b*, *hsa-miR-181c*, *hsa-miR-181d*, *hsa-miR-185*, *hsa-miR199a-5p*, *hsa-miR-200a*, *hsa-miR-328*, *hsa-miR-340*, *hsa-miR-383*, *hsa-miR-455*, and *hsa-miR-496* in DU-145 cells transfected with control vector or pCMV-ROR2 vector were determined by RT-qPCR. Expression of *U6* snRNA was used as loading control.

**Supplemental Figure 10. Lower ROR2 level correlated to worse survival and higher recurrence in PCa patients.** Kaplan-Meier plot was used to demonstrate the correlation between ROR2 level in prostate tumor and disease-free survival time of PCa patients in Sboner Rubin Prostate GSE 16560 (N=253 for high ROR2 expression and N=28 for low ROR2 expression) (A), cancer recurrence status after treatment in Taylor MSKCC Prostate database (N=93 for high ROR2 expression and N=47 for low ROR2 expression) (B), and TCGA-PARD dataset (N=199 for high *ROR2* gene level and N=300 for low *ROR2* gene level) (C). Datasets were extracted and analyzed from SurvExpress (<http://bioinformatica.mty.itesm.mx:8080/Biomatec/SurvivaX.jsp>). The *p* value smaller than 0.05 was considered as statistically significant.

**Supplemental Table 1.** **Primer sequence.** Sequence of primers used for qRT-PCR analysis was listed.

**Supplemental Table 2. Antibody information.** All antibodies used for Micro-Western Array or Western blotting assay were listed.
