# Supplementary material for: ROR2 suppresses metastasis of prostate cancer via regulation of miR-199a-5p–PIAS3–AKT2 signaling axis
Source: Cell Death Dis. 2020 May 15;11(5):376. doi: 10.1038/s41419-020-2587-9 (PMC7228945; doi:10.1038/s41419-020-2587-9)
Supplement: Supplementary file 2 — Supplemental Material and Methods [file 41419_2020_2587_MOESM2_ESM.docx]

**Supplemental Materials and Methods**

**Quantitative real-time PCR for human PCa cDNA Array**

The total RNA was isolated by using Qiagen RNeasy Mini kit (Qiagen, Venlo, Netherlands), and 2 μg complementary DNA was produced by RevertAid H Minus First Strand cDNA kit (Thermo Fisher Scientific). The ROR2 mRNA level of human prostate tissues and different Gleason score of PCa was determined on TissueScan Prostate Tissue qPCR Array HPRT101~103 (OriGene Technologies, Rockville, MD, USA) according to the manufacturers’ instruction with Maxima SYBR Green/ROX qPCR Master Mix (2x) (Fermentas, Glen Burnie, MA, USA) and analyzed by Applied Biosystems 7500 Real-Time PCR system (Thermo Fisher Scientific). The expression level of mRNA was normalized with GAPDH. All primers used in present study were listed at supplemental Table 1.

**Public domain data**

Data were downloaded from Oncomine database (http://www.oncomine.com). Expression profile of *ROR2* gene was extracted from LaTulippe prostate dataset (Human Genome U95A-Av2 Array), which contained 3 prostate glands, 23 primary prostate tumors and 9 metastatic prostate tumors^1^. Expression profile of *ROR2* gene was extracted from Grasso prostate dataset (Agilent Human Genome 44K), which contained 51 primary prostate tumors and 32 metastatic prostate tumors^2^. Expression profiles of *ROR2* genes expression analysis was extracted from Chandran prostate dataset (GSE6752) (Affymetrix GeneChip HGU95av2, HGU95b and HGU95c arrays), which contained 10 primary prostate tumors and 21 metastatic prostate tumors^3^. Expression profiles of *ROR2* gene expression analysis was extracted from Ramaswamy prostate dataset (HumanGeneFL Array, Hu35KsubA Array), which contained 10 primary prostate tumors and 4 metastatic prostate tumors^4^. Expression profiles of *ROR2* gene expression analysis was extracted from Ramaswamy prostate 2 dataset (HumanGeneFL Array, Hu35KsubA Array), which contained 10 primary prostate tumors and 3 metastatic prostate tumors^5^. Expression profiles of *ROR2* gene from Taylor prostate 3 dataset was detected by Agilent 244K array comparative genomic hybridization (aCGH) microarrays, which contained 29 prostate gland, 131 prostate carcinoma and 19 metastatic prostate cancers^6^. Expression profile of *ROR2* gene was extracted from Varambally prostate dataset (Human Genome U133 Plus 2.0 Array), which contained 7 primary prostate tumors and 6 metastatic prostate tumors^7^. Expression profiles of *ROR2* gene expression analysis was performed using Affymetrix U95A-Av2 human gene arrays with 8,603 features for individual gene/expressed sequence tag clusters extracted from Yu prostate, which contained 23 prostate gland, 64 prostate carcinoma and 25 metastatic prostate cancer from patients received radical prostatectomy^8^.

Clinical significance of *ROR2* in PCa patients was analyzed from SurvExpress (http://bioinformatica.mty.itesm.mx:8080/Biomatec/SurvivaX.jsp) online database. Correlation of expression level of ROR2 and patient’s survival rate was analyzed from Sboner Rubin Prostate GSE16560 under maximize risk groups condition. Correlation of expression level of ROR2 and patients recurrence free status was analyzed from Taylor MSKCC Prostate dataset under maximize risk groups condition^9^.

**Reference**

1 LaTulippe, E. *et al.* Comprehensive gene expression analysis of prostate cancer reveals distinct transcriptional programs associated with metastatic disease. *Cancer research* **62**, 4499-4506 (2002).

2 Grasso, C. S. *et al.* The mutational landscape of lethal castration-resistant prostate cancer. *Nature* **487**, 239-243 (2012).

3 Chandran, U. R. *et al.* Gene expression profiles of prostate cancer reveal involvement of multiple molecular pathways in the metastatic process. *BMC Cancer* **7**, 64 (2007).

4 Ramaswamy, S. *et al.* Multiclass cancer diagnosis using tumor gene expression signatures. *Proc Natl Acad Sci U S A* **98**, 15149-15154 (2001).

5 Ramaswamy, S., Ross, K. N., Lander, E. S. & Golub, T. R. A molecular signature of metastasis in primary solid tumors. *Nat Genet* **33**, 49-54 (2003).

6 Taylor, B. S. *et al.* Integrative Genomic Profiling of Human Prostate Cancer. *Cancer cell* **18**, 11-22 (2010).

7 Varambally, S. *et al.* Integrative genomic and proteomic analysis of prostate cancer reveals signatures of metastatic progression. *Cancer cell* **8**, 393-406 (2005).

8 Yu, Y. P. *et al.* Gene expression alterations in prostate cancer predicting tumor aggression and preceding development of malignancy. *Journal of Clinical Oncology* **22**, 2790-2799 (2004).

9 Taylor, B. S. *et al.* Integrative genomic profiling of human prostate cancer. *Cancer Cell* **18**, 11-22 (2010).
