# Supplementary material for: ROR2 suppresses metastasis of prostate cancer via regulation of miR-199a-5p–PIAS3–AKT2 signaling axis
Source: Cell Death Dis. 2020 May 15;11(5):376. doi: 10.1038/s41419-020-2587-9 (PMC7228945; doi:10.1038/s41419-020-2587-9)
Supplement: Supplementary file 3 — Supplemental Table 1 [file 41419_2020_2587_MOESM3_ESM.docx]

| Gene name | primer sequence |
| --- | --- |
| GAPDH | ACAGTCAGCCGCATCTTCTT (forward) |
| GAPDH | ACGACCAAATCCGTTGACTC (reverse) |
| PIAS3 | GAGCCGACATCCAAGGTTTA (forward) |
| PIAS3 | CAAGGGCATCCTGTTCATCT (reverse) |
| ROR2 | CCTGGTGCTTTACGCAGAATA (forward) |
| ROR2 | TGGGGACCAAGATGTACAGAA (reverse) |
| U6 snRNA | CTCGCTTCGGCAGCACAT (forward) |
| U6 snRNA | TTTGCGTGTCATCCTTGCG (reverse) |
| has-miR-1 | TGGAATGTAAAGAAGTATGT |
| has-miR-18a | AGGTGCATCTAGTGCAG |
| has-miR-18b | AGGTGCATCTAGTGCAG |
| has-miR-141 | CTTCCAGTACAGTGTTGG |
| has-miR-143 | GCAGTGCTGCATCTCTG |
| has-miR-150 | TCTCCCAACCCTTGTAC |
| has-miR-181a | TTCAACGCTGTCGGTGA |
| has-miR-181b | TTCATTGCTGTCGGTGG |
| has-miR-181c | CATTCAACCTGTCGGTG |
| has-miR-181d | CATTCATTGTTGTCGGTG |
| has-miR-185 | AGAGAAAGGCAGTTCCTG |
| has-miR-199a-5p | CCAGTGTTCAGACTACC |
| has-miR-200a | CTTACCGGACAGTGCTG |
| has-miR-328 | CCTCTCTGCCCTTCCG |
| has-miR-340 | TATAAAGCAATGAGACTGAT |
| has-miR-383 | TCAGAAGGTGATTGTGGC |
| has-miR-455 | GTGCCTTTGGACTACATC |
| has-miR-496 | GAGTATTACATGGCCAATC |
| has-miR-613 | GGAATGTTCCTTCTTTGC |
| miRNA reverse primer | GAACATGTCTGCGTATCTC |
