# Supplementary material for: ROR2 suppresses metastasis of prostate cancer via regulation of miR-199a-5p–PIAS3–AKT2 signaling axis
Source: Cell Death Dis. 2020 May 15;11(5):376. doi: 10.1038/s41419-020-2587-9 (PMC7228945; doi:10.1038/s41419-020-2587-9)
Supplement: Supplementary file 4 — Supplemental Table 2 [file 41419_2020_2587_MOESM4_ESM.docx]

| **Antibody list** |  |  |
| --- | --- | --- |
| Antibodies | Companies | Catalog No. |
| α-tubulin | Origene | TA307175 |
| β-actin | Novus | NB600-501 |
| β-catenin | cell signaling | #9582 |
| ABCG2 | abcam | ab108312 |
| ABCG5 | abcam | ab124965 |
| Akt | cell signaling | #9272 |
| Akt1 | millipore | 05-796 |
| Akt2 | Novus | NBP1-26594 |
| Akt3 | millipore | 07-383 |
| Akt3 | millipore | 05-780 |
| c-Myc | abcam | ab32072 |
| CKⅠ | abcam | ab108296 |
| CKⅡα | abcam | ab76040 |
| CKⅡβ | abcam | ab133576 |
| Claudin3 | Novus | NBP1-67517 |
| Claudin4 | Novus | NB100-91712 |
| Dkk1 | abcam | ab109416 |
| Dvl-1 | Santa Cruz | sc-8025 |
| Dvl-2 | cell signaling | #3216 |
| Dvl-3 | Santa Cruz | sc-8027 |
| E-cadherin | BD | 610182 |
| E-cadherin | abcam | ab133597 |
| FAK | cell signaling | #3285 |
| FAK | abcam | ab40794 |
| DDK tag (Flag) | Novus | NB600-345 |
| DDK tag (Flag) | Origene | TA50011-100 |
| FZD2 | Genetex | GTX88690 |
| FZD4 | R&D | MAB194 |
| FZD5 | millipore | 06-756 |
| FZD7 | Genetex | GTX88831 |
| GAPDH | Novus | NB300-322 |
| GSK3α | cell signaling | #4337 |
| GSK3β | cell signaling | #9315 |
| ILK | abcam | ab52480 |
| Jagged1 | cell signaling | #2620 |
| JNK | cell signaling | #9258 |
| KDM4C | Santa Cruz | sc-98678 |
| KLF4 | Santa Cruz | sc-20691 |
| Lamin A/C | Genetex | GTX111677 |
| LRP5 | Genetex | GTX60567 |
| LRP6 | Genetex | GTX111104 |
| MMP9 | abcam | ab76003 |
| NF-kB p50 | millipore | 04-234 |
| NF-kB p65 | cell signaling | #6956 |
| NF-kB p65 | millipore | 04-235 |
| NF-kB p65 | Thermo | RB-9034-R7 |
| N-cadherin | BD | 610920 |
| Nanog | cell signaling | #4903 |
| Nanog | cell signaling | #3580 |
| Oct4 | cell signaling | #2750 |
| p-Akt (S474) | cell signaling | #9271 |
| p-Akt (T308) | cell signaling | #9275 |
| p-β-catenin (S37) | abcam | ab75777 |
| p-β-catenin (T41/S45) | abcam | ab81305 |
| p-Dvl-2 (S143) | abcam | ab124933 |
| p-Dvl-2 (T224) | abcam | ab124941 |
| p-FAK (Y397) | abcam | ab81298 |
| p-FAK (Y576/577) | abcam | ab76244 |
| p-FAK (Y861) | millipore | 07-832 |
| p-GSK3α (S21) | cell signaling | #9316 |
| p-GSK3β (S9) | cell signaling | #9322 |
| PIAS1 | abcam | ab109388 |
| PIAS2 | abcam | ab126601 |
| PIAS3 | abcam | ab22856 |
| PIAS3 | cell signaling | #9042 |
| PKM2 | cell signaling | #4053 |
| p-JNK (T183/Y185) | cell signaling | #4668 |
| p-Rac1 (S71) | cell signaling | #2461 |
| p-Smad3 (S423/S425) | abcam | ab52903 |
| p-Stat3 (Y705) | cell signaling | #9145 |
| Rho | abcam | ab40673 |
| ROR1 | R&D | AF2000 |
| ROR2 | biorbyt | orb38364 |
| ROR2 | Santa Cruz | sc-374174 |
| Slug | cell signaling | #9585 |
| Slug | Novus | NBP2-27182SS |
| Smad2 | abcam | ab33875 |
| Smad2 | abcam | ab40855 |
| Smad3 | abcam | ab40854 |
| Smad4 | abcam | ab40759 |
| Snail | cell signaling | #3879 |
| SOX2 | cell signaling | #3579 |
| Sp1 | abcam | ab124804 |
| Stat3 | cell signaling | #9139 |
| SUMO2/3 | abcam | ab109005 |
| SUMO4 | abcam | ab126606 |
| twist1 | Genetex | GTX127310 |
| Vimentin | abcam | ab92547 |
| WIF1 | abcam | ab155101 |
| Wnt1 | Santa Cruz | sc-5630 |
| Wnt3a | Santa Cruz | sc-136163 |
| Wnt5a/b | cell signaling | #2530 |
| Wnt5a | Santa Cruz | sc-365370 |
| YAP | abcam | ab52771 |
