# Supplementary figures and images for: ROR2 suppresses metastasis of prostate cancer via regulation of miR-199a-5p–PIAS3–AKT2 signaling axis
Source: Cell Death Dis. 2020 May 15;11(5):376. doi: 10.1038/s41419-020-2587-9 (PMC7228945; doi:10.1038/s41419-020-2587-9)

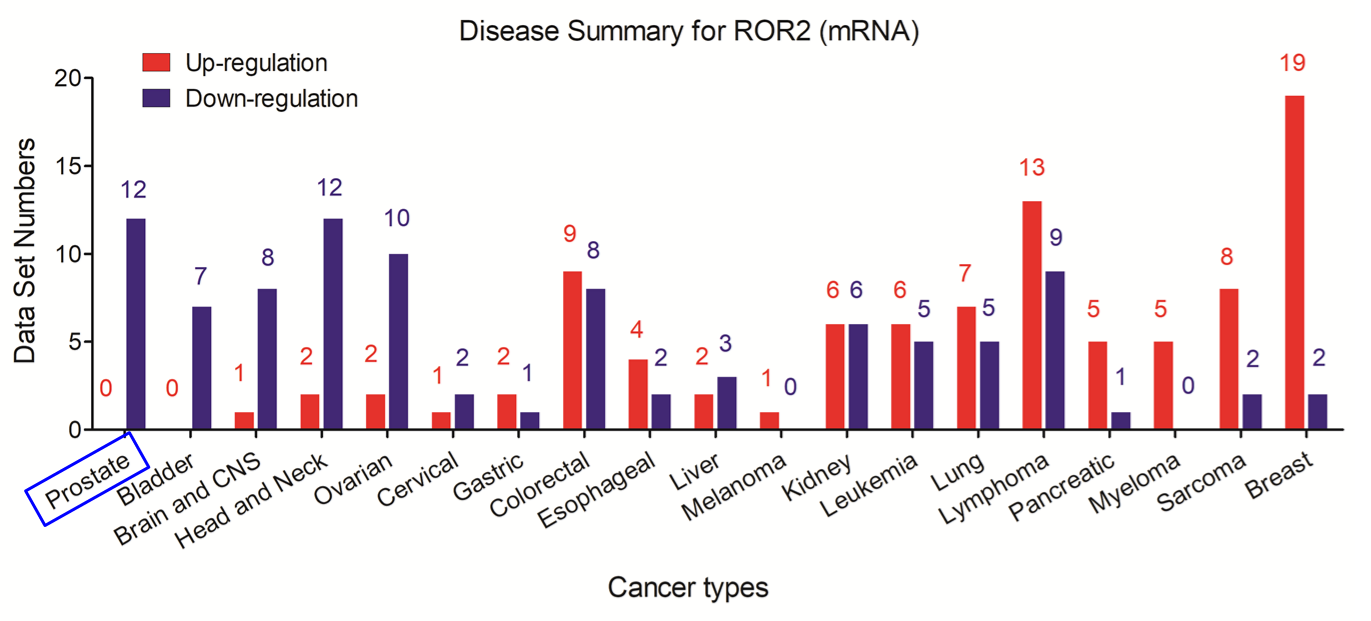

Supplement: Supplementary file 5 — Supplemental Figure 1 [file 41419_2020_2587_MOESM5_ESM.tif]

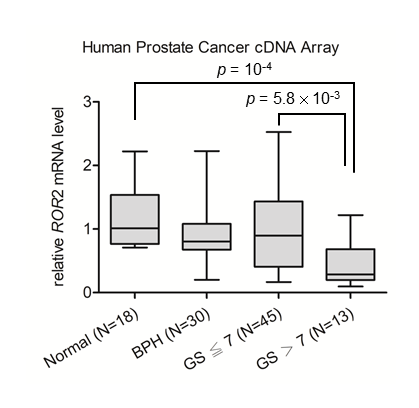

Supplement: Supplementary file 6 — Supplemental Figure 2 [file 41419_2020_2587_MOESM6_ESM.tif]

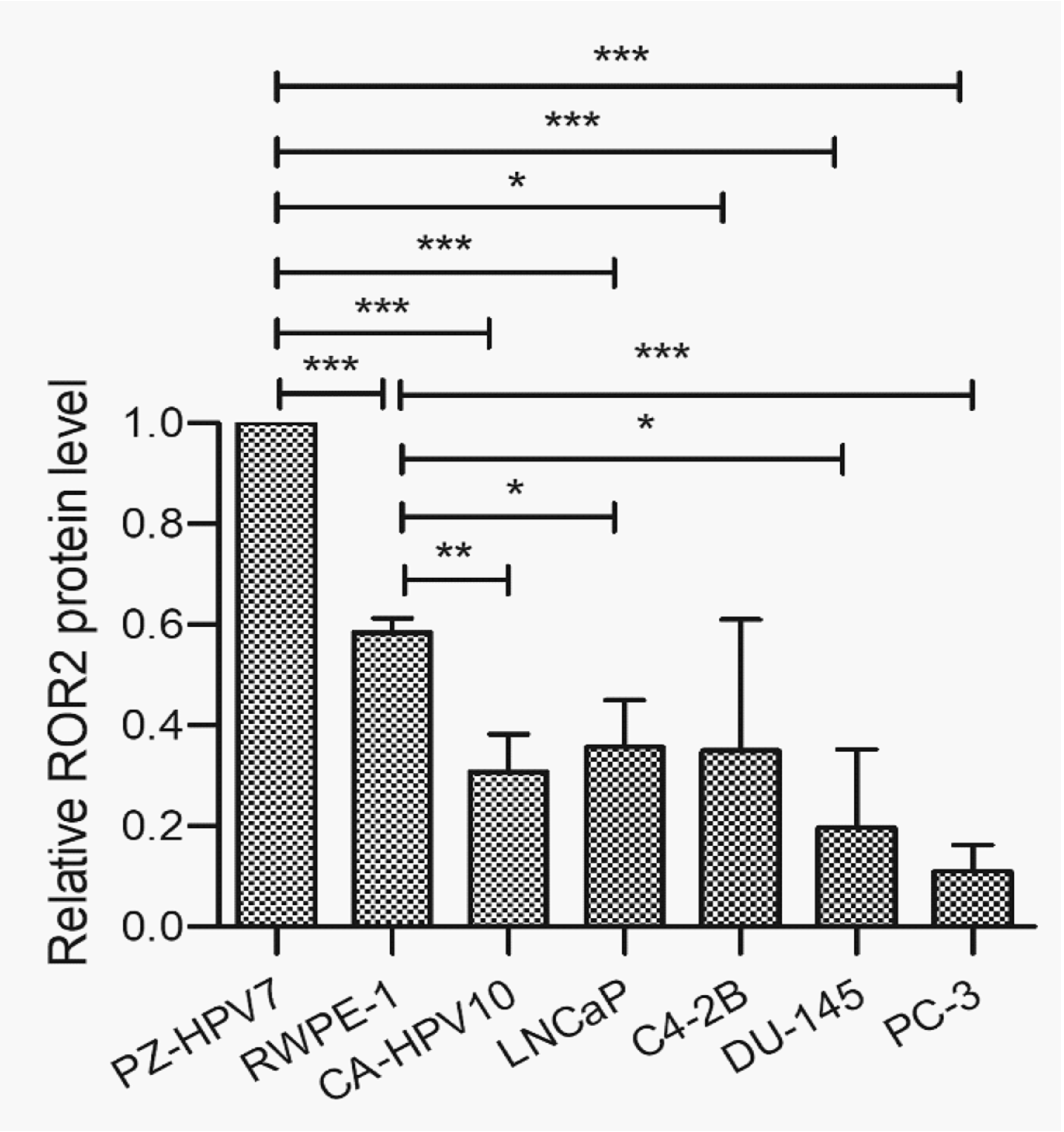

Supplement: Supplementary file 7 — Supplemental Figure 3 [file 41419_2020_2587_MOESM7_ESM.tif]

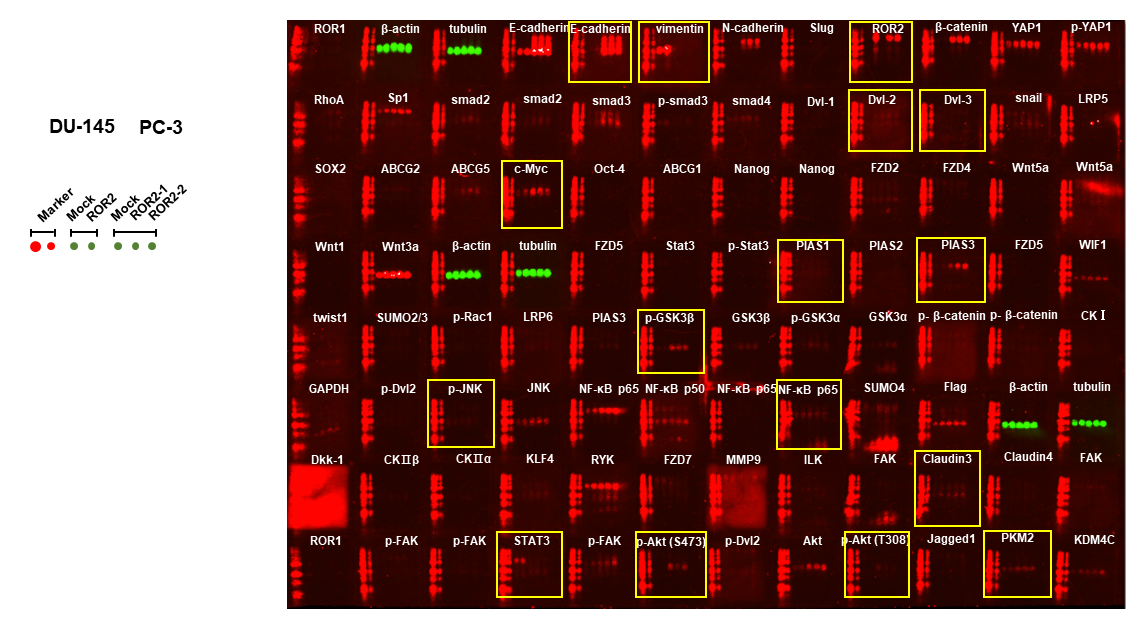

Supplement: Supplementary file 8 — Supplemental Figure 4 [file 41419_2020_2587_MOESM8_ESM.tif]

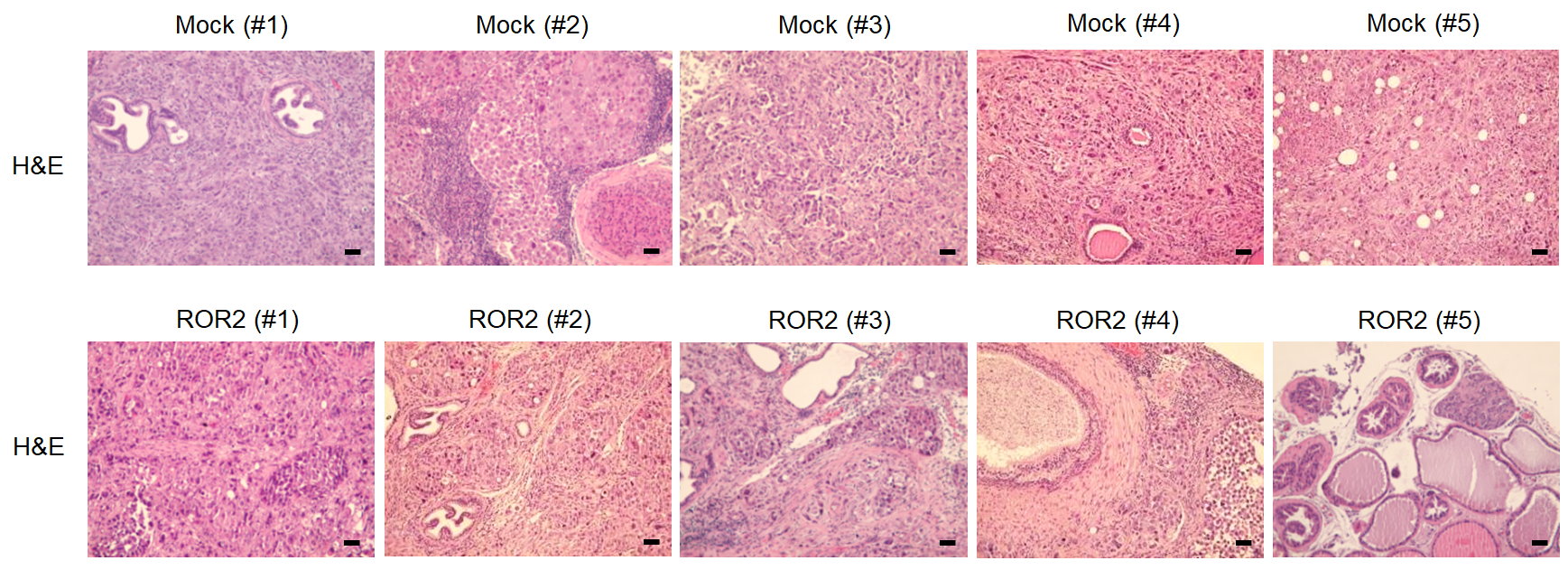

Supplement: Supplementary file 9 — Supplemental Figure 5 [file 41419_2020_2587_MOESM9_ESM.tif]

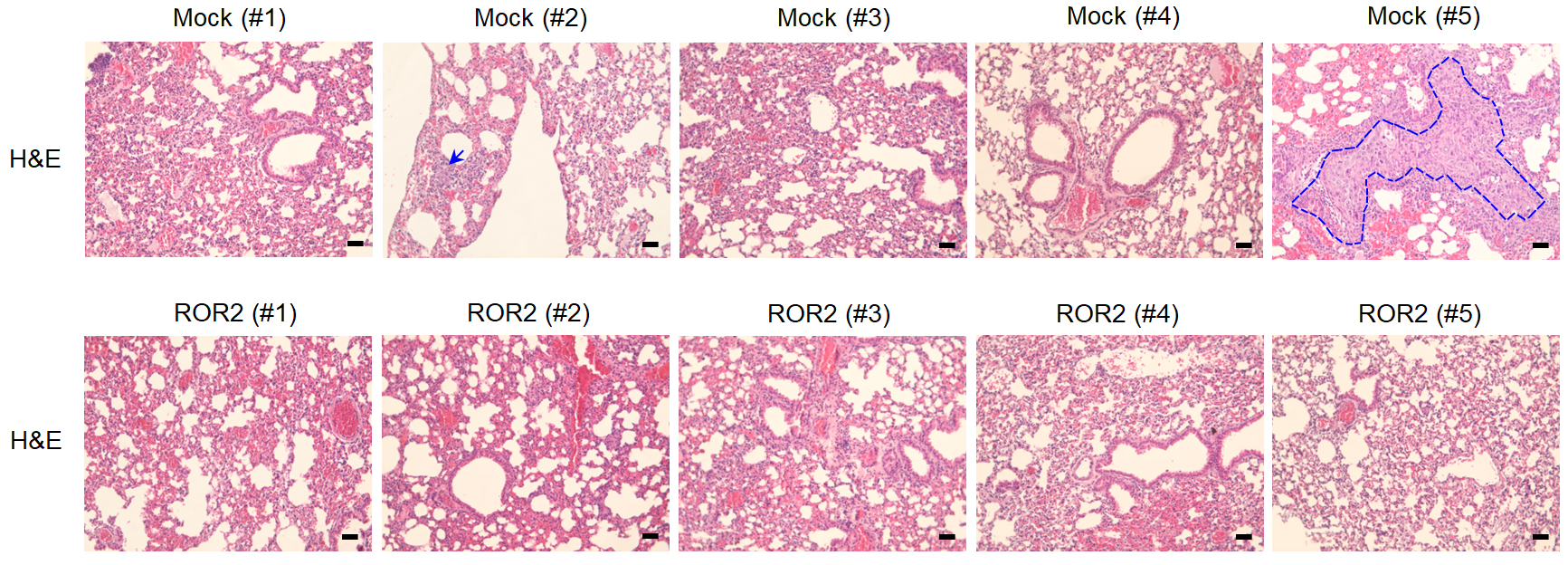

Supplement: Supplementary file 10 — Supplemental Figure 6 [file 41419_2020_2587_MOESM10_ESM.tif]

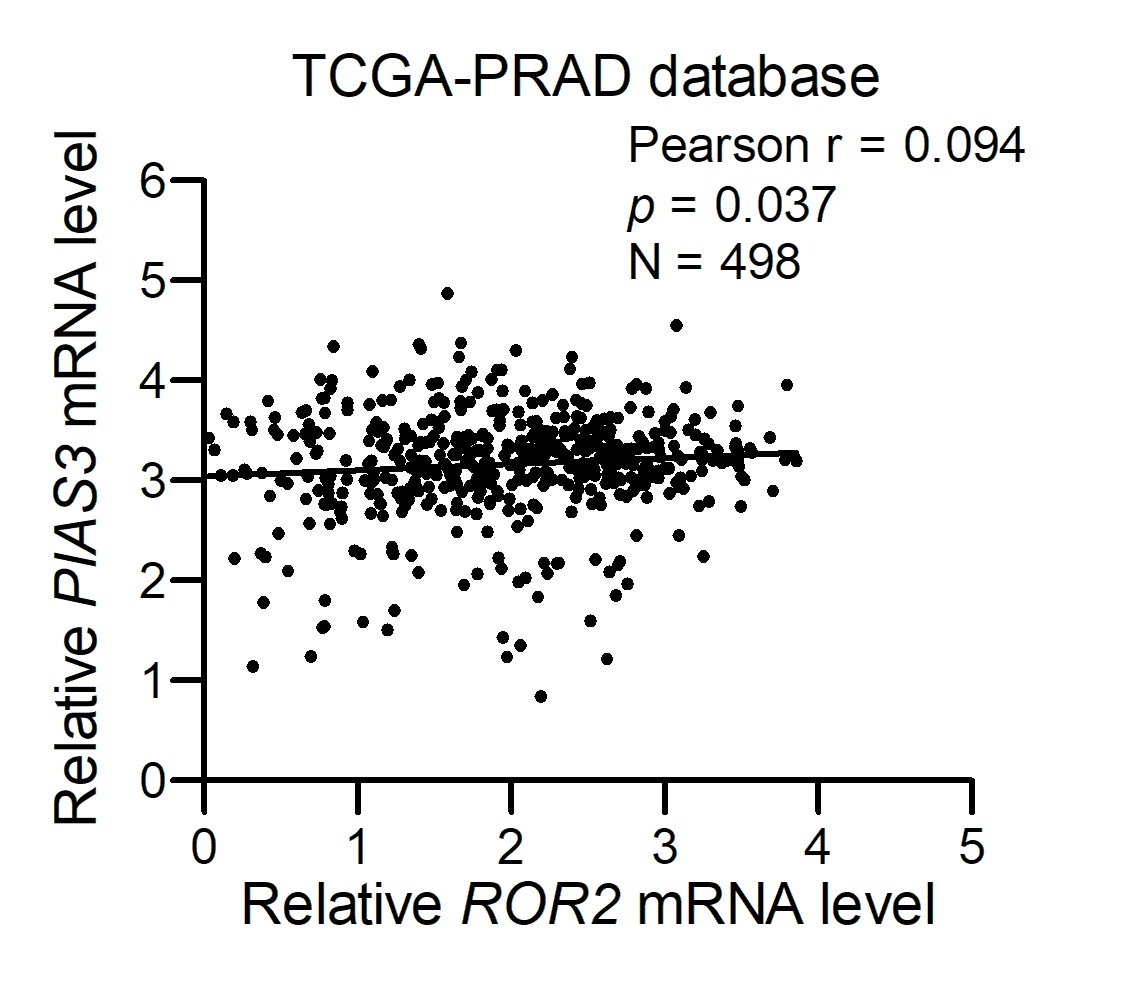

Supplement: Supplementary file 11 — Supplemental Figure 7 [file 41419_2020_2587_MOESM11_ESM.tif]

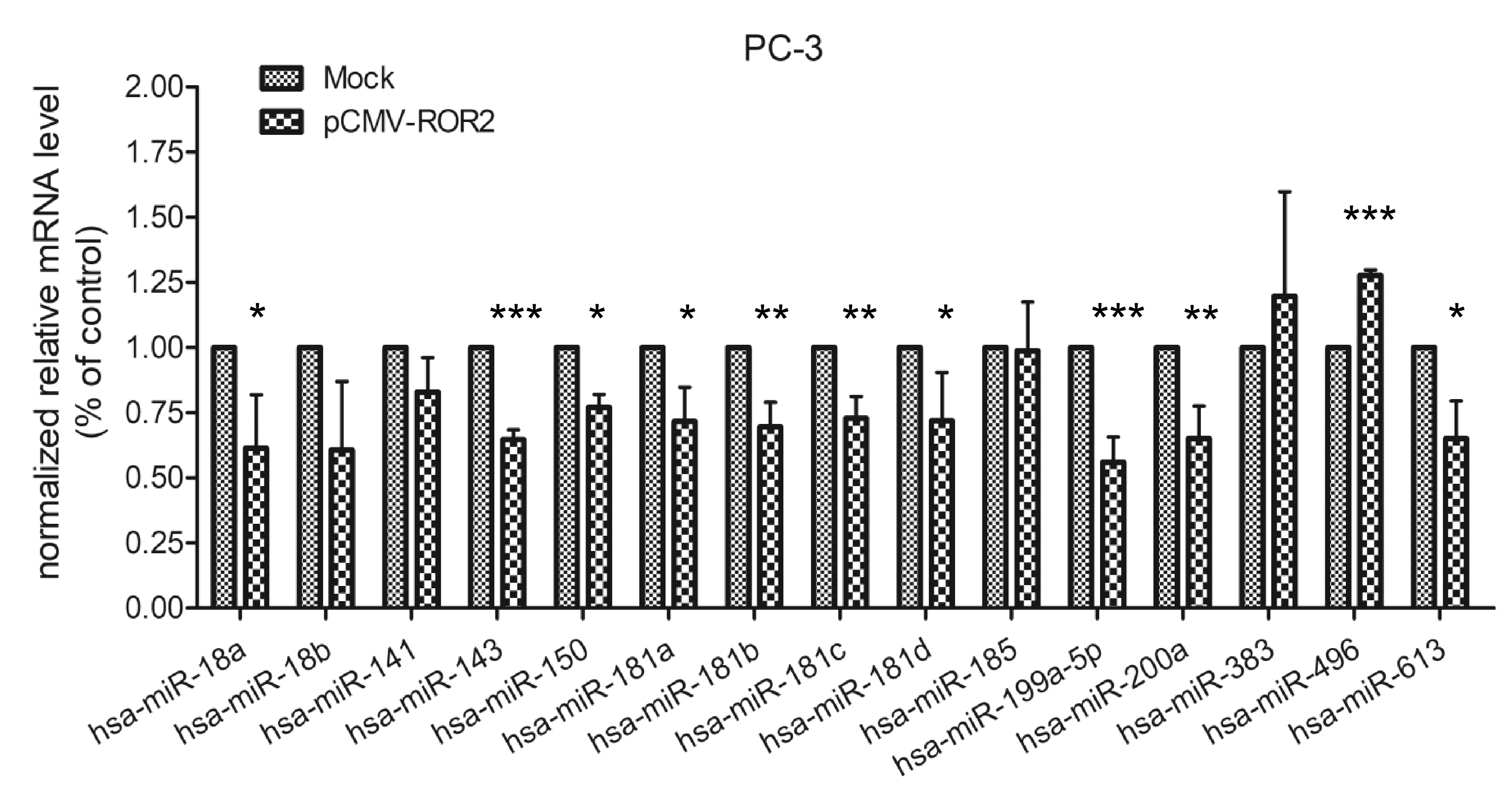

Supplement: Supplementary file 12 — Supplemental Figure 8 [file 41419_2020_2587_MOESM12_ESM.tif]

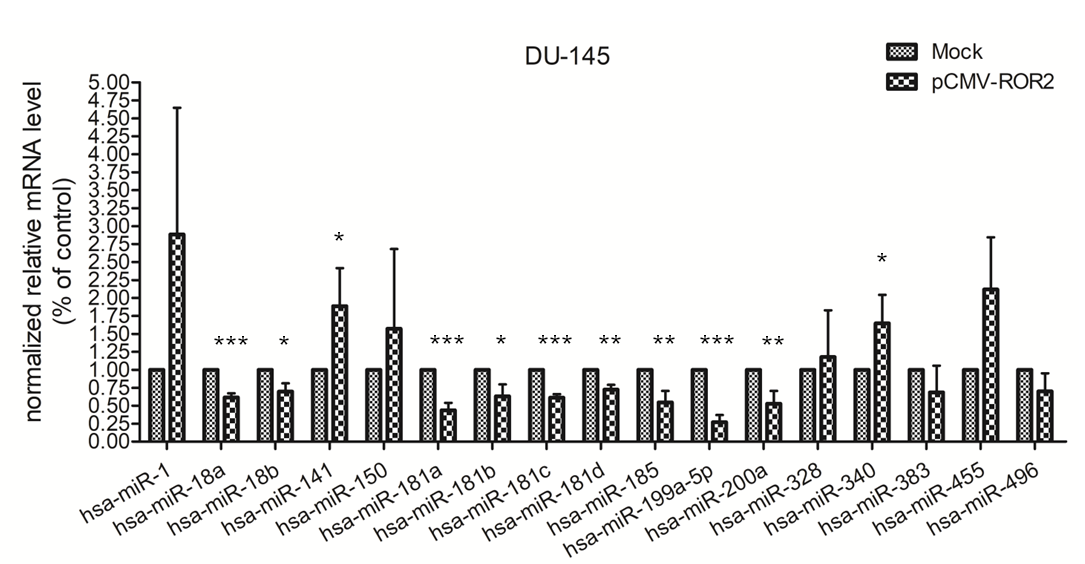

Supplement: Supplementary file 13 — Supplemental Figure 9 [file 41419_2020_2587_MOESM13_ESM.tif]

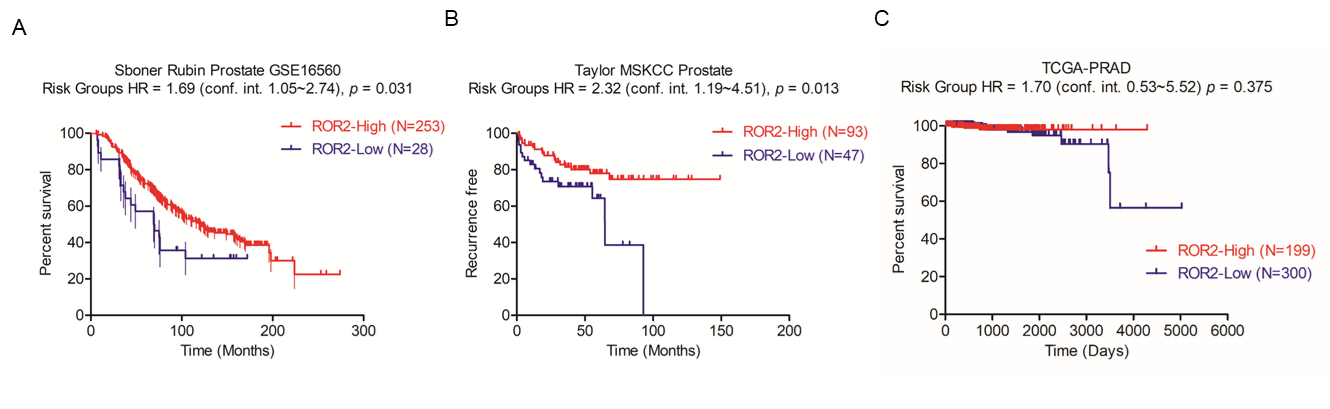

Supplement: Supplementary file 14 — Supplemental Figure 10 [file 41419_2020_2587_MOESM14_ESM.tif]
